# Forensic Medicine and Toxicology

**Published:** 1877-12

**Authors:** 


### Books Reviewed.

*Forensic Medicine and Toxicology.* By W. Bathurst Woodman, M. D., F.R.C.P. and Charles Meymott Tidy, M. B., F.C.S. Philadelphia: Lindsay & Blakiston, 1877. Buffalo: T. H. Butler. pp. 1083. Price, cloth $7.50. Leather, $8.50.

This is the joint production of two most favorably known authors. The work that they have already given the profession would lead us to expect something more than common, and we were not disappointed. Nearly one half the volume is devoted to Toxicology, and is one of the most complete treatises upon this subject in our language. It is fully illustrated with eight full page lithographic plates and over one hundred wood cuts. These illustrations are so skillfully drawn that lawyers and non-professional men can easily master the main facts.

It is preeminently English, and modern to the last degree, but the authors have laid all countries and writers under contribution in order to produce the present excellent volume. We quote from the *London Lancet* to show the opinion in which it is held in England. "It contains almost everything that can be found in other works on the subject; but it is no mere compilation. Dr. Woodman and Dr. Tidy have both thought out the subject for themselves, and, with care, industry, and acumen, have brought together a mass of facts, which is little short of astounding. The book is worthy to take its place alongside of any work on the same subject, and must prove of great use to all who practice in criminal courts, and to all medical practitioners. We have no hesitation in recommending it to our readers."

We can only echo this just opinion of so excellent a production. C.

